# Computational modeling predicts the ionic mechanism of late-onset responses in unipolar brush cells

**DOI:** 10.3389/fncel.2014.00237

**Published:** 2014-08-20

**Authors:** Sathyaa Subramaniyam, Sergio Solinas, Paola Perin, Francesca Locatelli, Sergio Masetto, Egidio D'Angelo

**Affiliations:** ^1^Neurophysiology Unit, Department of Brain and Behavioral Science, University of PaviaPavia, Italy; ^2^Consorzio Interuniversitario per le Scienze Fisiche della Materia (CNISM)Pavia, Italy; ^3^Neurophysiology Unit, Brain Connectivity Center, Istituto Neurologico IRCCS C. MondinoPavia, Italy

**Keywords:** biorealistic modeling, unipolar brush cells, vestibular cerebellum, slow synaptic responses, ionic channel regulation

## Abstract

Unipolar Brush Cells (UBCs) have been suggested to play a critical role in cerebellar functioning, yet the corresponding cellular mechanisms remain poorly understood. UBCs have recently been reported to generate, in addition to early-onset glutamate receptor-dependent synaptic responses, a late-onset response (LOR) composed of a slow depolarizing ramp followed by a spike burst (Locatelli et al., 2013). The LOR activates as a consequence of synaptic activity and involves an intracellular cascade modulating H- and TRP-current gating. In order to assess the LOR mechanisms, we have developed a UBC multi-compartmental model (including soma, dendrite, initial segment, and axon) incorporating biologically realistic representations of ionic currents and a cytoplasmic coupling mechanism regulating TRP and H channel gating. The model finely reproduced UBC responses to current injection, including a burst triggered by a low-threshold spike (LTS) sustained by CaLVA currents, a persistent discharge sustained by CaHVA currents, and a rebound burst following hyperpolarization sustained by H- and CaLVA-currents. Moreover, the model predicted that H- and TRP-current regulation was necessary and sufficient to generate the LOR and its dependence on the intensity and duration of mossy fiber activity. Therefore, the model showed that, using a basic set of ionic channels, UBCs generate a rich repertoire of bursts, which could effectively implement tunable delay-lines in the local microcircuit.

## Introduction

The classical neurotransmission process is based on release of a chemical transmitter followed by activation of ionotropic receptors and the consequent generation of membrane potential changes in the postsynaptic neuron (Katz and Miledi, 1964). In addition to this, slow neurotransmission mechanisms have been reported, which involve the activation of intracellular second-messenger cascades and slow regulation of membrane ionic channels. These can either be voltage-dependent channels taking part to regulation of neuronal electroresponsiveness, or be ionic cyclic nucleotide-gated channels (CNGCs) specifically involved in neuromodulatory responses. While these latter are well known in heart cells and photoreceptors, they have only recently been recognized in neurons (Brown et al., 1981; Wainger et al., 2001; Clapham, 2003). Interestingly, the unipolar brush cells (UBCs) of the cerebellum expresses both responses generated by ionotropic receptors (Rossi et al., 1995; van Dorp and De Zeeuw, 2014) and a late-onset response (LOR) generated by receptor coupling through cytoplasmic messengers to membrane ionic channels (Locatelli et al., 2013).

UBCs are interneurons of the cerebellar granular layer forming a complex network with mossy fibers, granule cells, Golgi cells, and other UBCs (Mugnaini et al., 1997). UBCs have been proposed to play an essential role for shifting and converting the phase of mossy fiber activity required for controlling and consolidating motor learning (Gao et al., 2012). When injected with depolarizing currents from a negative membrane potential, UBCs respond with a calcium spike priming a sodium spike burst (Russo et al., 2007). Then, as depolarizing currents are increased, the initial burst is followed by a tonic discharge (Diana et al., 2007). The ionic currents responsible for UBC electroresponsiveness have been partly clarified, including CaLVA and CaHVA currents, the H-current and different types of K currents (Diana et al., 2007). When activated synaptically, the LOR was shown to depend on the intracellular cAMP cascade and on subsequent regulation of H- and TRP-currents (Locatelli et al., 2013). Despite these observations, it remained unclear whether the known set of UBC ionic currents was sufficient to explain membrane electrogenesis, including responses to current injection and the LOR, and whether an intracellular messenger could effectively couple the neurotransmission process to LOR generation.

In this paper, intrinsic UBC electroresponsiveness was reproduced in a biologically realistic UBC multi-compartmental model. The model could accurately predict all known aspects of UBC electroresponiveness demonstrating the central role of H- and Ca^2+^ currents. The model then effectively predicted the LOR, when a generic intracellular mechanism was used to couple mossy fiber stimulation to TRP and H channel gating. LOR delay, duration and spike frequency turned out to be efficiently regulated by the intensity and duration of synaptic stimulation. Therefore, the model confirmed that a fundamental set of ionic channels was sufficient to explain both UBC electroresponsiveness and the LOR. This mechanism could contribute to generate tunable delay-lines in the cerebellar network (Kennedy et al., 2014).

## Methods

This work presents a combined modeling (I) and experimental analysis (II) of UBC electroresponsiveness, in which the models was accurately matched to biological responses (III). The model was written in NEURON (Hines et al., 2007; Davison et al., 2009).

### The UBC model

**Figure 1:**
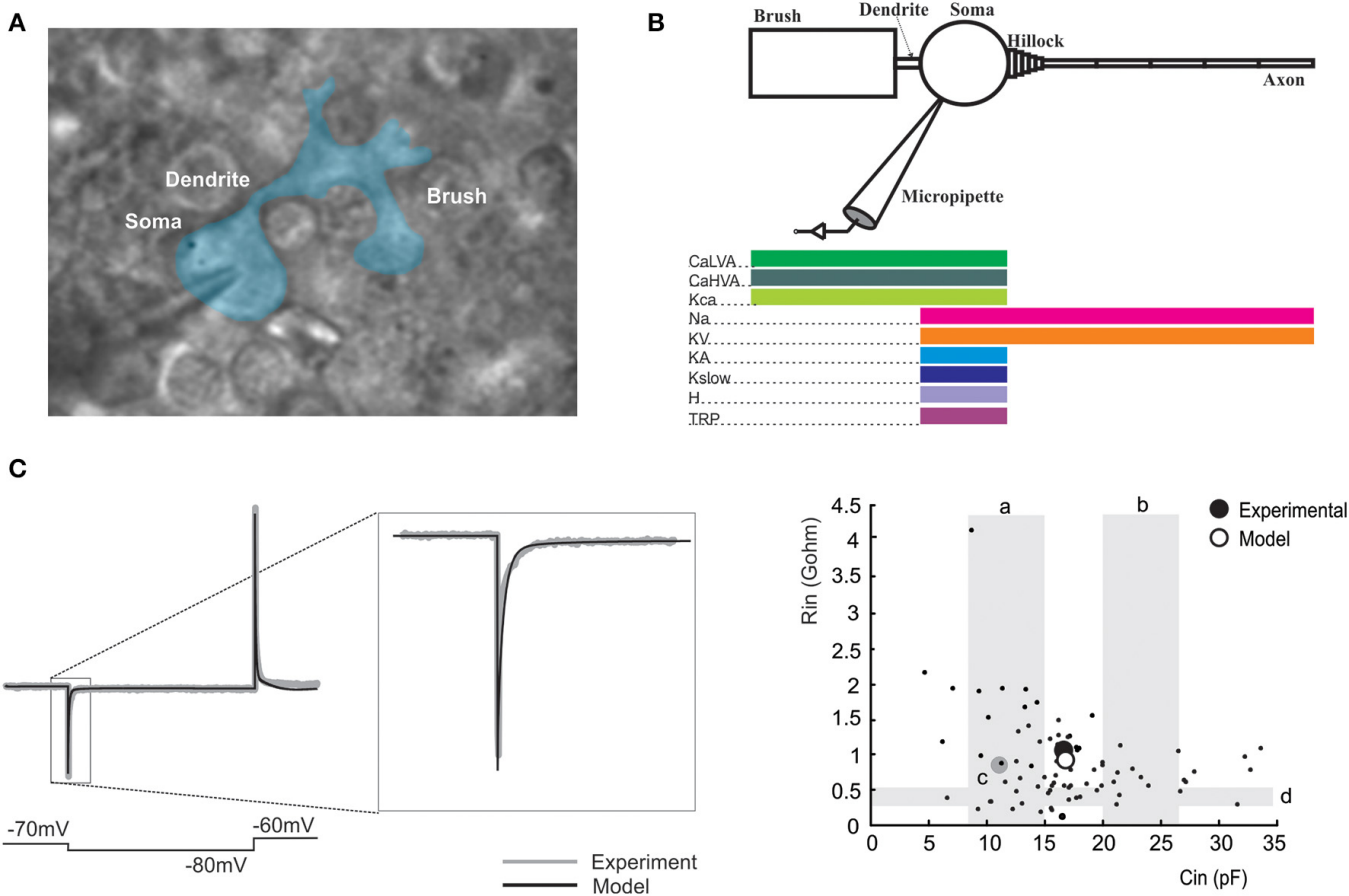
**UBC morphology and passive properties. (A)** A UBC recorded in patch-clamp whole-cell configuration and observed in transmitted light microscopy. The cell contour has been identified and color-filled. The different parts of the cell have been identified and labeled. **(B)** Morphology and distribution of ionic channels in the UBC model. Same color code as in **Figures 3–6, 8. (C)** The trace shows a voltage-clamp recording of a current transient elicited in a UBC by a −10 mV voltage step (blue trace) and the corresponding model reconstruction using the same protocol (red trace). The transient is enlarged in the inset to show the close adherence of model to experimental responses. The plot shows the relationship between input resistance (R_in_, GΩ) and input capacitance (C_in_, pF) calculated from the fitting of current transients, both in experiments and in the model. Experimental points were taken from the UBC population recorded for this paper (*n* = 30) and from Locatelli et al. (2013); unpublished, *n* = 51. The model prediction falls in the middle of the experimental data distribution. The values range reported by other works are shown as gray shadowed areas: *a* (Diana et al., 2007), *b* (Russo et al., 2007, 2008), *c* (Locatelli et al., 2013), *d* (Birnstiel et al., 2009).

A multi-compartmental model representative of the main morphological components of a UBC was constructed using the NEURON simulator (NEURON version 7.3; Hines and Carnevale, 2001). The model consisted of soma, dendrite, initial segment and axon compartments generating a morpho-electrical equivalent of the UBC (Figure 1A). The voltage- and Ca^2+^-dependent mechanisms (see below) were distributed among the compartments. With this approach, the model reproduced satisfactorily basic aspects of UBC electroresponsiveness elicited by somatic current injection. It should also be noted that, although the existence of UBC subtypes has been suggested based on histochemical analysis, the basic electroresponsive properties were homogeneous in a large majority of UBCs (Locatelli et al., 2013). Thus, we have reconstructed a canonical UBC model, which simulates the typical electrophysiological behavior of UBCs and lies within the scatter of physiological parameters values. The model was coupled to a recording electrode to reproduce realistic current-clamp conditions and was calibrated on the cellular response corrected for −10 mV liquid-junction potential.

The model ionic conductances were non-homogenously distributed over different compartments (Figure 1B; Table 1). Gating kinetics were corrected using a Q_10_ = 5 for *I_CaLVA_* activation (Destexhe et al., 1994) and a Q_10_ = 3 for all the other currents according to the relation Q^(Tsim−Texp)/10^_10_ (Gutfreund et al., 1995) to account for temperature differences between experimental recordings and the model (30°C). Nernst equilibrium potentials were pre-calculated from ionic concentrations used in current-clamp recordings and maintained fixed, except for the Ca^2+^ equilibrium potential, which was updated during simulation according to the Goldman-Hodgkin-Katz (GHK) equation. The maximum ionic conductances were regulated to fit the UBC responses to various stimulations (Traub and Llinás, 1979; Traub et al., 1991; Vanier and Bower, 1999; Achard and De Schutter, 2006; Druckmann et al., 2007, 2008, 2011, 2013; Solinas et al., 2007a,b). Ionic channel gating was modeled following the Hodgkin and Huxley formulation or Markov-chains for multi-state transitions using mathematical methods reported previously (D'Angelo et al., 2001; Nieus et al., 2006; Solinas et al., 2007a,b) and are reported in Table 1. Membrane voltage was obtained as the time integral of the equation (Yamada et al., 1989).

(1) $$\frac{dV}{dt}=-\frac{1}{C_{m}}\;*\left\{\sum \left[g_{i}\;*\left(V-V_{i}\right)\right]+i_{inj}\right\}$$

**Table 1:** **Ionic mechanisms in the model**.

**Conductance state variables**		**n**	**Gmax (S/cm^2^)**	**Vrev (mV)**	**α (s^−1^)**	**β (s^−1^)**	**References**
g_Na_	Activation		0.192	63	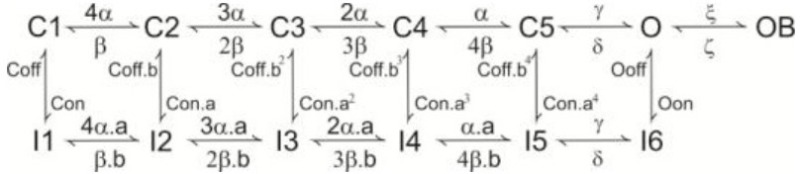		Raman and Bean, 2001
	Inactivation						
	Closed						
	Blocked						
g_KV_	Activation	3	6.75 × 10^−4^	−84.69	$\begin{array}{l} \;1/\left(1+\exp\left(-(v+24)/15.4\right)\right)\\ \tau_{m}=1000*\left(3.4225e-5+0.00498*\exp\left(-\frac{v}{-28.29}\right)\right)*3\\ \;0.31+0.69/\left(1+\exp\left((v+5.802)/11.2\right)\right)\\ \;\tau_{h}=1000*(0.0012+0.0023*\exp(-0.141*v)) \end{array}$		Anwar et al., 2012
	Inactivation	1					
g_KA_	Activation	3	0.007	−84.69	$\begin{array}{l} 4.88826/\left[1+\exp\left(-(v+9.197)/23.327\right)\right]\\ 0.11/\left[\exp\left(\frac{v+111.33}{12.84}\right)+1\right]\\ x_{\infty }=1/\left(1+\exp\left(-(v+38)/17\right)\right) \end{array}$	$\begin{array}{l} \;0.99285/\left[\exp\left((v+18.2791)/19.4717\right)\right]\\ \;0.1/\left[\exp\left(-\frac{v+49.95}{8.9}\right)+1\right]\\ \;y_{\infty }=1/\left(1+\exp\left((v+78.8)/8.4\right)\right) \end{array}$	D'Angelo et al., 2001; Nieus et al., 2006
	Inactivation	1					
g_KCa_	Activation	1	0.0052	−84.69	$2.5/\left(1+\left(1.5e-3*\frac{\exp\left(-\frac{v}{11.765}\right)}{\left[Ca\right]}\right)\right)$	$1.5/\left(1+\left(\frac{\left[Ca\right]}{1.5e-4*\exp\left(-\frac{v}{11.765}\right)}\right)\right)$	D'Angelo et al., 2001; Nieus et al., 2006
g_Kslow_	Activation	1	0.0008	−84.69	0.0033 * exp((*v* + 30)/40)	0.0033 * exp (−(*v* + 30)/20)	D'Angelo et al., 2001; Nieus et al., 2006
					*x*_∞_ = 1/[1 + exp(−(*v* + 45)/6)]		
g_CaHVA_	Activation	2	2.15 × 10^−4^	129.33	0.04944 * exp((*v* + 29.06)/15.873)	0.04944 * exp ((*v* + 29.06)/15.873)	(D'Angelo et al., 2001; Nieus et al., 2006)
	Inactivation	1			0.0013 * exp (−(*v* + 48)/18.183)	0.0013 * exp (−(*v* + 48)/18.183)	
							Kinetics adapted to fit; (Diana et al., 2007)
g_CaLVA_	Activation	2	6.3 × 10^−5^ (cm/s)	129.33	$\;\begin{array}{l} \;1/(1+\exp((v+52)/-5))\\ \tau_{m}=(1+\frac{1}{\frac{\exp(v+40)}{9}}+\exp((v+102)/-18))\\ \begin{array}{l} \;1/(1+\exp((v+72)/7))\\ \;\tau_{h}=\left(15+1/\exp\left((v+32)/7\right)\right) \end{array} \end{array}$		(Anwar et al., 2012)
	Inactivation	1					Kinetics adapted to fit; (Diana et al., 2007)
*g_H_*		1	5.72 × 10^−4^	−30	l_∞_ = 1/(1 + exp((*v* + 91.5)/7.7))		(McCormick and Pape, 1990)
					τ_*l*_ = [exp (0.2 * 0.65 * (*v* + 91.5))]/(4.5 * 0.0005 * [1 + (exp(0.2 * (*v* + 91.5)))])		
							Kinetics adapted to fit; (Locatelli et al., 2013)

*The table reports the equations used to calculate membrane conductances used in the model. In most cases, the rate constants α_x_, β_x_, α_y_, and β_y_ were used to calculate x_∞_, y_∞_, τ_x_, and τ_y_ according to a Hodgkin-Huxley scheme (Hodgkin and Huxley, 1952),*
$$g_{i}=Gmax_{i}*x_{i}^{zi}*y_{i}$$
*where, Gmax_i_ is the maximum ionic conductance, x_i_ and y_i_ are state variables (probabilities ranging from 0 to 1) for a gating particle, and z_i_ is the number of such gating particles in ionic channel i. x and y (the suffix i omitted) were related to the first order rate constants α and ß by the equations*
$$x_{\infty }=\frac{\alpha_{x}}{\alpha_{x}+\beta_{x}},y_{\infty }=\frac{\alpha_{y}}{\alpha_{y}+\beta_{y}};\;\tau_{x}=\frac{1}{\left(\alpha_{x}+\beta_{x}\right)},\tau_{y}=\frac{1}{\left(\alpha_{x}+\beta_{x}\right)}$$

*Where α and β are functions of voltage. The state variable kinetics were*
$$\frac{dx}{dt}=\frac{\left(x_{\infty }-x\right)}{\tau_{x}},\frac{dy}{dt}=\frac{\left(y_{\infty }-y\right)}{\tau_{y}}$$

*In some cases, the equations for x_∞_, y_∞_, τ_x_, or τ_y_ are reported directly. Two exceptions to this general scheme descend from the availability of advanced ionic channel models. The CaLVA channel was built using the GHK equation (Anwar et al., 2012) and ionic permeability. The Na channel was built using a 13-state Markov-chain scheme (Raman and Bean, 2001; Magistretti et al., 2006) yielding the probability of channel opening. Eventually, both permeability and probability of channel opening were transformed into ionic conductances and used to calculate the model currents*.

Where *V* is membrane potential, *C_m_* membrane capacitance, *g_i_* are ionic conductances and *V_i_* reversal potentials (the subscript *i* indicates different channels), and *i_inj_* is the injected current. The intracellular Ca^2+^ concentration, [Ca^2+^], was calculated through the equation,

(2) $$\frac{d\left[Ca^{2+}\right]}{dt}=\frac{-I_{Ca}}{\left(2F\;*\;Ad\right)}-\beta_{Ca}\left(\left[Ca^{2+}\right]-{\left[Ca^{2+}\right]}_{0}\right)$$

Where *d* is the depth of a shell adjacent to the cell surface of area α, β_*Ca*_ determines the loss of Ca^2+^ ions from the shell approximating the effect of fluxes, ionic pumps, diffusion, and buffers (Traub and Llinás, 1979; McCormick and Huguenard, 1992; De Schutter and Smolen, 1998), and [*Ca*^2+^]_0_ is resting [*Ca*^2+^].

#### Morphology

Model morphology was reconstructed by keeping the dimensions of every compartment within the ranges reported in the literature. The model contains a spherical cell body (8 μm diameter; Rossi et al., 1995; Morin et al., 2001; Kalinichenko and Okhotin, 2005; Russo et al., 2007), a short dendritic shaft (50 μm length and 2 μm diameter; Diño and Mugnaini, 2000) that originates from soma and connects to a comparatively large compartment named brush (25.25 μm length and 10 μm diameter; (Mugnaini et al., 1997, 2011) on its other end. The surface of the brush is extended by unfolding the three-dimensional structure of dendrites of real UBCs, which are enriched by many filopodia/dendrioles that form a structure reminiscent of a brush. The UBC axon was reproduced with five identical compartments (80 μm length and 0.5 μm diameter; Kalinichenko and Okhotin, 2005) that are serially connected to the axon initial segment (AIS). The AIS was made of 5 compartments of equal length (0.5 μm) and decreasing diameter moving toward the axon (from 3.2 to 0.8 μm; Figure 1B). The micropipette that was attached to the soma was simulated as a passive cable with sealed end, null capacitance, infinite wall resistance and an axial resistance (20 MΩ) equivalent to the sum of series and access resistance measured experimentally.

#### Passive properties

The axial specific resistance of axon and dendrites was set to 100 Ω cm, the specific membrane resistance was set to 47.6 KΩ /cm^2^ and specific membrane capacitance was set at 1 μF/cm^2^ (cf. Roth and Häusser, 2001; Diwakar et al., 2009, 2011). The input resistance (R_in_, GΩ) and input capacitance (C_in_, pF) of the model were calculated from the current transient elicited by 250 ms voltage step of ΔV = −10 mV from the holding potential of −70 mV (Figure 1C). The charge displacement during voltage steps followed a tri-phasic trajectory reflecting electrotonic compartmentalization, with the faster transient being related to the soma, the intermediate transient being mostly contributed by the brush and dendritic shaft and the slowest transient being mostly related to the axon (Locatelli et al., 2013); see Section II in Methods). Current transient analysis yielded R_in_ = 1.04 GΩ and C_in_ = 16.5 pF. The C_in_ value corresponds closely to the total cell capacitance of 16.7 pF calculated by summing the capacitance of the soma (2 pF), brush and dendritic shaft (12.7 pF), and axon (2 pF).

#### Mechanisms of intrinsic electroresponsiveness

The mechanisms of intrinsic electroresponsiveness were based on experimental observations including current-clamp (Locatelli et al., 2013) and voltage-clamp recordings (Diana et al., 2007; Birnstiel et al., 2009; Locatelli et al., 2013). Most currents introduced into the model were based on experimental observations like *I_CaLVA_, I_CaHVA_, I_KCa_, I_H_, I_TRP_, I_Na_ while others including I_leak_, I_KV_, I_KA_, I_Kslow_* were introduced because needed to control the passive properties as well as the timing and frequency of spike discharge. In summary, the model included 10 defined ionic mechanisms (see Table 1 for their mathematical representation):

*I_leak_*, background leakage current.
*I_TRP_*, cyclic nucleotide gated leakage current.
*I_H_*, hyperpolarization activated cationic current (H-type).
*I_CaLVA_*, low-voltage activated calcium current (T-type).
*I_CaHVA_*, high-voltage activated calcium current (L-type).
*I_KCa_*, calcium-dependent K current current (BK-type).
*I_Na_*, Na current with transient, resurgent and persistent components.
*I_KV_*, delayed-rectifier K current.
*I_KA_*, fast-activating K current (A-type).
*I_Kslow_*, slow-activating K current (M-type).

***Leakage currents (*I_leak_*, *I_TRP_*)***. The model included a leakage current (*I_leak_*) adapted from a granule cell model (D'Angelo et al., 2001) and localized in all 13 compartments of the UBC model with a reversal potential at −70 mV. The corresponding conductance was tuned to obtain appropriate passive current transients. The presence of *I_TRP_* in UBCs was reported by Russo et al. (2007) and confirmed by Locatelli et al. (2013). TRP channels in UBCs are ruthenium red sensitive, voltage-independent cationic currents. TRP channels were modeled as a simple leakage channels with a reversal potential at 0 mV (Petersson et al., 2011). The model was adapted to change its conductance in response to second messenger concentration (see below).

***H current (*I_H_*)***. The presence of *I_H_* in UBC was reported by Russo et al. (2007) and confirmed by Locatelli et al. (2013). *I_H_* in UBCs is manifest during the application of hyperpolarizing current steps causing sagging inward rectification (Santoro et al., 2000). *I_H_* proved able to slowly depolarize the UBC over the whole subthreshold range and causing a steady depolarization (~4 mV) between −50 and −70 mV (Locatelli et al., 2013). After regulating current densities to match the sag and the steady-state level of hyperpolarizing responses, the model generated a robust rebound depolarization, as observed experimentally. The *I_H_* model was adapted from the HCN current in thalamo-cortical neurons (McCormick and Pape, 1990) and was tuned using experimental data on maximum conductance and channel kinetics in UBCs (Locatelli et al., 2013). The reconstructed *I_H_* showed a 10% activation at −108 mV and half-activation at −91.5 mV with a slope factor of 7.7 mV. The *I_H_* activation time constant was mono-exponential and decreased toward negative membrane potentials with mean values ranging from ~180 ms at −130 mV to ~630 ms at −90 mV, in keeping with experimental results (Locatelli et al., 2013). The model was adapted to shift its activation curve in reaction to second messenger concentration (see below).

***Na currents (I_Na_)***. UBCs express TTX sensitive voltage-gated channels with transient, persistent and resurgent current components (Afshari et al., 2004). The Na channels of these neurons tend to recover from inactivation through open states, giving rise to a “resurgent” current that flows upon repolarization from positive potentials (Raman and Bean, 1997; Afshari et al., 2004). The recovery from inactivation is associated with the resurgent current, which shortens the refractory period between action potentials favoring the generation of high-frequency bursts (Raman and Bean, 2001; Khaliq et al., 2003). As the resurgent properties are typical of Nav1.6 (Raman and Bean, 1997; Afshari et al., 2004), we adopted the corresponding granule cell model (Magistretti et al., 2006). These channels have been inserted in soma, hillock and axonal compartments according to the most common localization found in other neurons, e.g., granule cells (Diwakar et al., 2009) and Purkinje cells (Palmer et al., 2010).

***K currents (I_KV_, I_KA_, I_Kslow_, I_KCa_)***. Multiple classes of potassium channels (K^+^) are inserted in the model. K^+^ delayed rectifier (KV), the fast inactivating A-type (KA), the persistent M-type (Kslow), along with the Ca^2+^-dependent K channels (BK-type) were adapted without any modifications from the granule cell model (D'Angelo et al., 2001; Nieus et al., 2006). A-type and BK-type calcium dependent potassium channels were localized in brush, dendrites (McKay et al., 2005) and soma compartments with different maximum conductances to regulate first spike delay, spike after-hyperpolarization (AHP) and interspike interval. KV was localized in soma, hillock and axonal segments to regulate spike AHP. A slow repolarizing M-type (*I_Kslow_*) and a fast-activating A-type (*I_KA_*) K currents in UBCs cells have not been reported yet, but were needed to regulate spike delay and tonic discharge frequency.

***Ca currents (I_CaLVA_, I_CaHVA_)***. In UBC two types of calcium channels are expressed and play an important role in controlling electroresponsiveness. UBCs are bimodal, as they can either fire high-frequency spike burst when stimulated from hyperpolarized potentials or generate tonic discharge during sustained depolarization. Fast inactivating Ca^2+^ channels (CaLVA or T-type) generate low-threshold spikes (LTS) triggering the high-frequency spike bursts, while poorly-inactivating Ca^2+^ channels (CaHVA or L-type) sustain the tonic firing. T-type calcium channels in UBCs are of the Ca3.1 subtype (Hildebrand et al., 2009). Channel kinetics were adapted from a previous model (Anwar et al., 2012) in order to reconstruct UBC specific current kinetics, voltage-dependence and time-dependence (Diana et al., 2007; Birnstiel et al., 2009). L-type calcium currents were also reconstructed adapting a previous model (D'Angelo et al., 2001) accounting for UBC specific properties (Diana et al., 2007; Birnstiel et al., 2009). In the case of T-type channels, the ionic conductance was computed using GHK equation and the Ca^2+^ permeability (Hirschberg et al., 1998; Anwar et al., 2012). Both types of calcium channels were localized in brush, dendritic shaft and soma compartments. Maximum conductance of T-type channels was set at a higher level in brush compared to soma, whereas that of L-type channels was maintained the same in all compartments (Diana et al., 2007; Birnstiel et al., 2009).

***Ca^2+^ dynamics***. Ca^2+^ dynamics were adapted to yield Ca^2+^ transients of about 1 μM following spike-dependent opening of CaHVA channels (Gabbiani et al., 1994). Parameters used in Equation (6) (*d* = 200 nm, β_Ca_ = 1.5, and [Ca^2+^]_0_ = 100 nM) were similar to those used in other neurons (e.g., Traub and Llinás, 1979; Traub, 1982; De Schutter and Smolen, 1998; D'Angelo et al., 2001). The computed intracellular Ca^2+^ concentration [*Ca*^2+^]_i_ activated *I_KCa_* just after the spike upstroke and generated the fast AHP. It should be noted that, in the model, *I_CaLVA_* was not allowed to contribute to the Ca^2+^ pool controlling *I_KCa_*.

***Special issues in modeling UBC electroresponsiveness***. In UBCs, *I_H_*, *I_CaLVA_*, and *I_CaHVA_* provided strong constraints for model tuning, since their conductances were known from experimental measurements. (i) After setting morphology and passive properties, *I_H_* was added with known conductance and its effect on the hyperpolarizing responses was evaluated. (ii) Then, *I_CaLVA_* and *I_CaHVA_* were added respecting the conductance, localization and proportions determined experimentally. (iii) The complement of spike-related currents was reconstructed with mechanisms taken from a previous granule cell model (D'Angelo et al., 2001; Nieus et al., 2006). The density of the Na^+^ current was adjusted to reproduce the action potential threshold and overshoot. The delayed rectifier K^+^ current, *I_KV_*, was needed to generate spike repolarization and balanced with *I*_KCa_ to match the spike shape. The requirement of a Ca^2+^-dependent voltage-dependent K^+^ current, *I_KCa_*, emerged from the inability of *I_KV_* to fully account for the fast phase of spike AHP and to guarantee Na^+^ channel re-priming at high discharge frequency. *I_KA_* was needed to regulate spike delay in response to depolarizing current injection and during rebound excitation and *I_Kslow_* was required to regulate tonic discharge frequency.

#### Receptor coupling to ionic channels: modeling the late-onset response

The LOR was reproduced in the model by modulating the *I_H_* and *I_TRP_* ionic currents with respect to the amount of intracellular factors related to the cAMP cascade (Locatelli et al., 2013). As a matter of fact, the exact intracellular-biochemical cascade driving the LOR is unknown. Therefore, we implemented a generic second messenger pathway reflecting general principles of biochemical organization (Wainger et al., 2001; Zhou et al., 2009; Shen et al., 2011). This includes a receptor *X* placed in proximity of the UBC synapse, which activates into X^*^ over a slow time course (>200 ms; consistent with Locatelli et al., 2013) upon mossy fiber bundle stimulation (Equation 3). Then X^*^ activates a second-messenger cascade transforming *Y* into Y^*^ (Equation 4). Finally, Y^*^ modulates the shift of *I_H_* activation curve and the TRP channel gating depolarizing the model and triggering the LOR. The cascade reaction is the following (see **Figure 4**):

(3) $$X\begin{array}{c} \alpha \\ ⇋\\ \beta \end{array}X^{*}$$

(4) $$Y\begin{array}{c} \delta (X^{*})\\ ⇋\\ \gamma \end{array}Y^{*}$$

Where,

(5) $$\alpha =\sum_{\text{s=1}}^{\text{nspk}}\Theta \left(t-t_{s}\right)\Theta \left(t_{s}+\tau -t\right)\omega$$

(6) $$\delta \left(X^{*}\right)=1/\text{​}\left(\exp\left(\left(X^{*}-X^{*}\_half\right)\text{​}/K\right)\right)$$

Where, α and β are the rates of forward and backward reactions. α is a step function that, at the time of occurrence (*t_s_*) of each input pulse, increases by the rate of synaptic weight (ω) and then decays back after a time interval τ (Equation 5). β is a backward rate constant that determines a fixed time to degrade X^*^ into *X*. The initial value of *X* was calibrated in order to reproduce the LOR generated by a 10-pulse burst at 100 Hz. Formation of Y^*^ from *Y* is a reversible reaction (Equation 6). The forward reaction increases Y^*^ depending on the amount X^*^. The initial *Y* value used in the model (5 mM) is in the range of the expected ATP level in neurons (Fitz, 2007). δ and γ determine the forward (*Y*→ Y^*)^ and backward (Y^*^ → *Y*) reaction rates, respectively. δ is a sigmoidal function, with half-activation X^*^_*half* = 2.7 mM and slope *K* = 0.75 mM^−1^.

The particle Y^*^ was used to modulate the H and TRP channels. In the case of TRP channels, the *G_max−TRP_* value was increased proportionally to Y^*^ (see **Figure 4**) (Equation 7). This mechanism is supported by observations of TRP channel modulation by intracellular cAMP (Petersson et al., 2011) and is consistent with the effects observed in UBCs (Locatelli et al., 2013). In the case of H channels, Y^*^ modified *I_H_* gating by proportionately shifting the steady-state activation curves to more positive potentials. This effect, observed in other cell types (DiFrancesco and Mangoni, 1994), is consistent with that observed in UBCs (see **Figure 4**; Locatelli et al., 2013). Thus, the H channel half-activation value was shifted proportionately to Y^*^ between −91.5 and −66.5 mV (Equation 8).

(7) $$new\;G_{max-TRP}=G_{max-TRP}\;*\;(0.16+Y^{*})$$

(8) $$new\;V_{inact-H}=V_{inact-H}+10\text{ ​}\left(\frac{1}{1+exp\left(0.6-Y^{*}/0.1\right)}\text{​}\right)$$

### Whole-cell recordings from UBCs in acute cerebellar slices

UBC recordings were carried out as reported previously (Locatelli et al., 2013). Whole-cell patch-clamp recordings were performed from 30 UBCs in the internal granular layer of lobule X in acute rat cerebellar slices of P18-P25 wistar rats. Out of these, 12 UBCs were used to study intrinsic electroresponsiveness and 18 to study the LOR. In addition, to estimate the passive properties and the resting membrane potential, data were also re-analyzed from a population of 51 UBCs reported by Locatelli et al. (2013) for a total of 81 measurements.

In brief, the patch pipettes were pulled with a horizontal puller (Sutter Instruments, Novato, CA, USA) from thick-walled borosilicate glass capillaries (Hingelberg, Germany) and had a resistance of 7–10 MΩ when filled with the intracellular solution (in mM): 126 potassium gluconate, 4 NaCl, 15 glucose, 5 Hepes, 1 MgSO4.7H2O, 0.1 BAPTA, 3 ATP, 100 μm GTP; pH adjusted to 7.2 with KOH (in solution at 0.1 mM). The BAPTA-Ca^2+^ buffer was prepared as explained previously (D'Angelo et al., 1995; Gall et al., 2003). To minimize pipette tip capacitance, pipettes were coated with Sylgard (Dow Chemical, Midland, MI, USA) and the bath level was kept as low as possible. After careful pipette capacitance cancelation (D'Angelo et al., 1995), −10 mV 250 ms voltage step were applied to the cell from the holding potential of −70 mV and the corresponding current transients were recorded (sampling frequency at 20–40 kHz, low-pass filtering 5 kHz). The current transient decayed multi-exponentially, probably reflecting charging of compartments corresponding to the UBC soma, dendritic shaft, brush, and axon. Tri-exponential fittings allowed us to estimate the electrode series resistance (*R_s_*) the cell input capacitance (*C_in_*) and the input resistance (*R_in_*) using the equation:

(9) $$\begin{array}{l} I(t)=A_{1}X\exp\left(k-t/\tau 1\right)+A_{2}X\exp\left(k-t/\tau 2\right)\\ \;+\;A_{3}X\exp\left(k-t/\tau 3\right)+C \end{array}$$

Series resistance (*R_s_*) was calculated as *R_s_* = τ_*VC*_*/C*_*in*_, where τ_*VC*_ = τ_1_ is the decay time constant of the current transient related to somatic charging. In our extended UBC sample, we measured *C_in_* = 16.9 ± 0.67 pF, *R_in_* = 0.87 ± 0.07 GΩ, *R_s_* = 22 ± 0.96 MΩ (mean ± MSE, *n* = 81 for all parameters).

All current-clamp recordings were performed using the fast current-clamp mode of the amplifier to accelerate membrane charging (D'Angelo et al., 1995, 1998; Prestori et al., 2008). The resting membrane potential was measured over 50 ms in 12 consecutive traces. Intrinsic excitability was investigated by setting resting membrane potential at −80 mV and injecting 800 ms current steps (from −16 to 48 pA in 4–8 pA increments). Current steps were applied from the holding potential of −80 mV and the corresponding voltage deflections were recorded (sampling frequency at 20–40 kHz, low-pass filtering 5 kHz).

Electrical stimulation was performed by placing a bipolar tungsten electrode over the mossy fiber bundle in the granular layer (D'Angelo et al., 1993, 1995). The stimuli consisted of voltage pulses (0.25 ms, 5–15 V) organized in 100 Hz trains of varying intensity and length.

### Data analysis and model matching to experimental data

#### Spike analysis

The analysis of experimental and simulated traces was performed using identical measurement procedures and the process was automated using dedicated scripts written in MATLAB (MathWorks, Natick, MA, USA). In response to depolarizing currents, spike threshold was detected at the point where the depolarization rate reached 5 mV/ms (Forti et al., 2006). For each spike, the overshoot and its position were measured. The *frequency ratio* (*instantaneous frequency*/*steady-state frequency*) in response to depolarizing currents was calculated as the ratio between the first and fourth ISI, which are representative of the burst and tonic discharge regions, respectively. The sag amplitude was calculated as the difference between the minimum and steady-state value of membrane potential during responses to hyperpolarizing currents. When the LOR was elicited, its *delay*, *duration* and all the firing parameters were measured. Data are reported as mean ± s.e.m.

#### Model construction, tuning, validation

The modeling procedure could be divided into three phases: *construction, tuning, and validation*. In the UBC model, after defining the morphological and passive properties, setting the ionic channel complement and the ionic channel gating properties (*construction*), the only free parameters remained the maximum ionic conductances. These were pre-adapted based on experimental estimates (see **Figure 3**) and then fine-tuned against experimental data using voltage traces elicited in response to step-current injection (pulses from different holding potentials and responses to hyperpolarization) (*tuning*). Finally, the model was used to predict response properties, which were not included in the data-set used for construction (*validation*). Model validation was performed at several levels by evaluating the matching with:

- Passive properties of a UBC population more extended than the one used to construct the model,
- Active electroresponsive properties comprising resting membrane potential, transition from burst to tonic firing, sagging responses to hyperpolarization, rebound excitation and burst inactivation from depolarized membrane potentials,
- LOR properties including dependence on the input pattern, intensity of the LOR generating currents, LOR pharmacological sensitivity.

#### Model robustness

As quantitative validation, we assessed the ability of the model in maintaining typical UBC properties once one of the maximum ionic conductances was varied in turn (Solinas et al., 2007a,b). Model robustness was evaluated both for eletroresponsiveness (see **Figure 6A**), for the LOR (see **Figure 6B**), and for their combination (**Figure 6C**).

## Results

### UBC multi-compartmental model: channel distribution and passive properties

UBCs (Figure 1A) were recorded from the granular layer of lamina X of the rat vestibular cerebellum (Mugnaini and Floris, 1994) using patch-clamp techniques in acute slices and were modeled using the NEURON simulator. In order to investigate whether the proposed LOR mechanisms were consistent with current knowledge on UBC intrinsic electroresponsiveness and whether the prediction of LOR generating mechanisms was correct (Locatelli et al., 2013), we have developed a detailed UBC computational model, in which synaptic transmission was coupled to H- and TRP-channels through a cytoplasmic modulatory mechanism.

**Figure 2:**
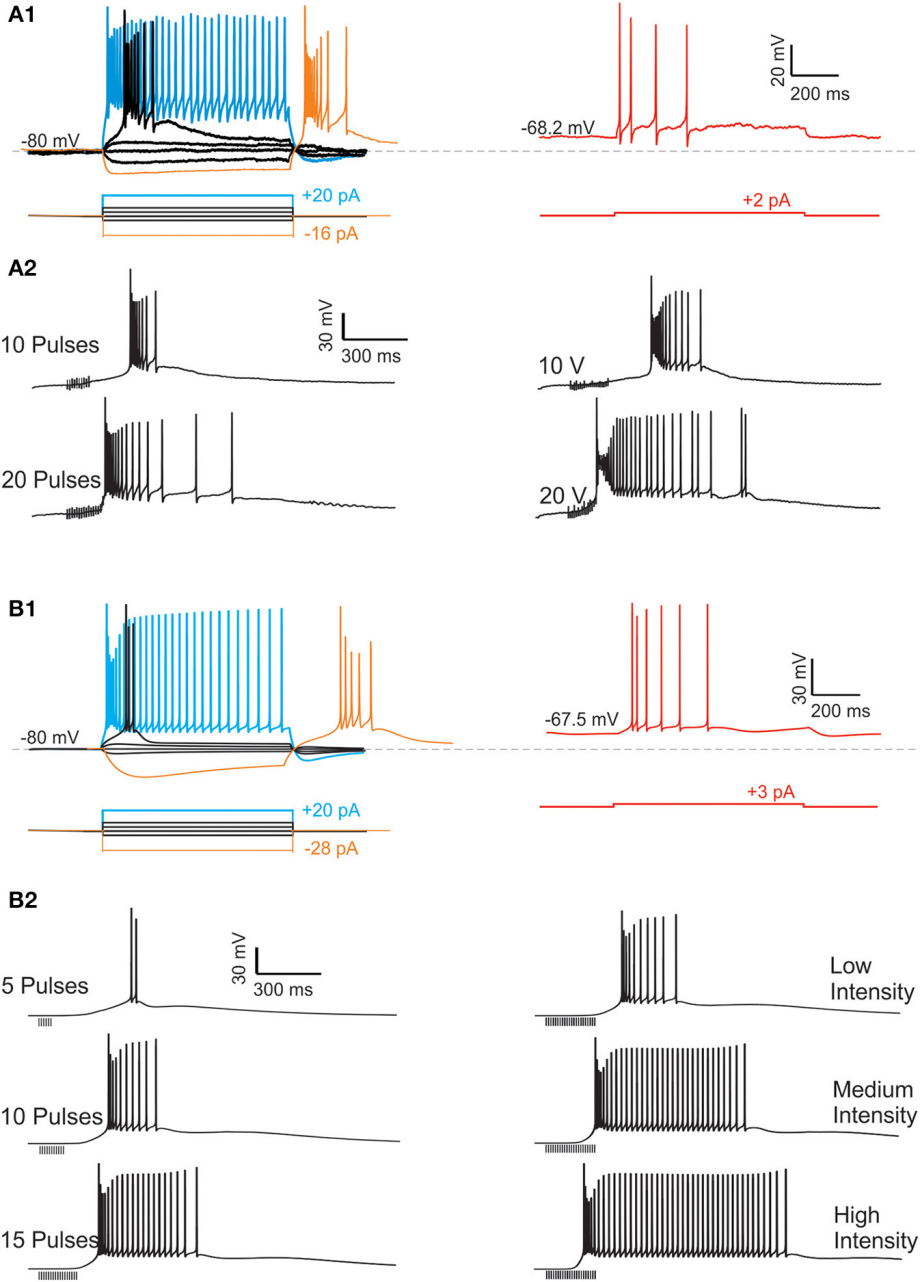
**Recordings and simulations of UBC responses**. The experimental responses **(A)** were taken from a representative cell maintained in current-clamp at the indicated holding potential and stimulated either with current pulses or with mossy fiber bundle stimulation. Identical stimulation patterns were used for the model **(B)**. **(A_1_)** The traces on the *left* show the responses of a UBC maintained at −80 mV and injected with currents steps of different intensity (protocol below the voltage traces). Depolarizing current steps of adequate intensity generate a low-threshold spike (LTS) surmounted by a burst of sodium spikes. At higher intensity, the burst is followed by a tonic action potential discharge. With hyperpolarizing current steps, the UBC response shows a sag at the beginning of the step and a rebound burst at the end of the step. The trace on the *right* shows the responses of the same UBC maintained at −68.2 mV to a depolarizing current step. In this case the LTS and the associated burst almost disappear. **(A_2_)** The traces on the *left* show the LOR evoked in the UBC [same as in **(A_1_)**] by mossy fiber bundle stimulation with pulse-trains of different length. The traces on the *right* show the LOR evoked by mossy fiber bundle stimulation with pulse-trains of different intensity (in V). (**B_1_,B_2_**) The traces show the responses of the UBC model corresponding to those generated in the experimental recordings shown in **(A_1_,A_2_)**. Note that the intensity of stimulation is in arbitrary units (low, medium, and high).

**Figure 3:**
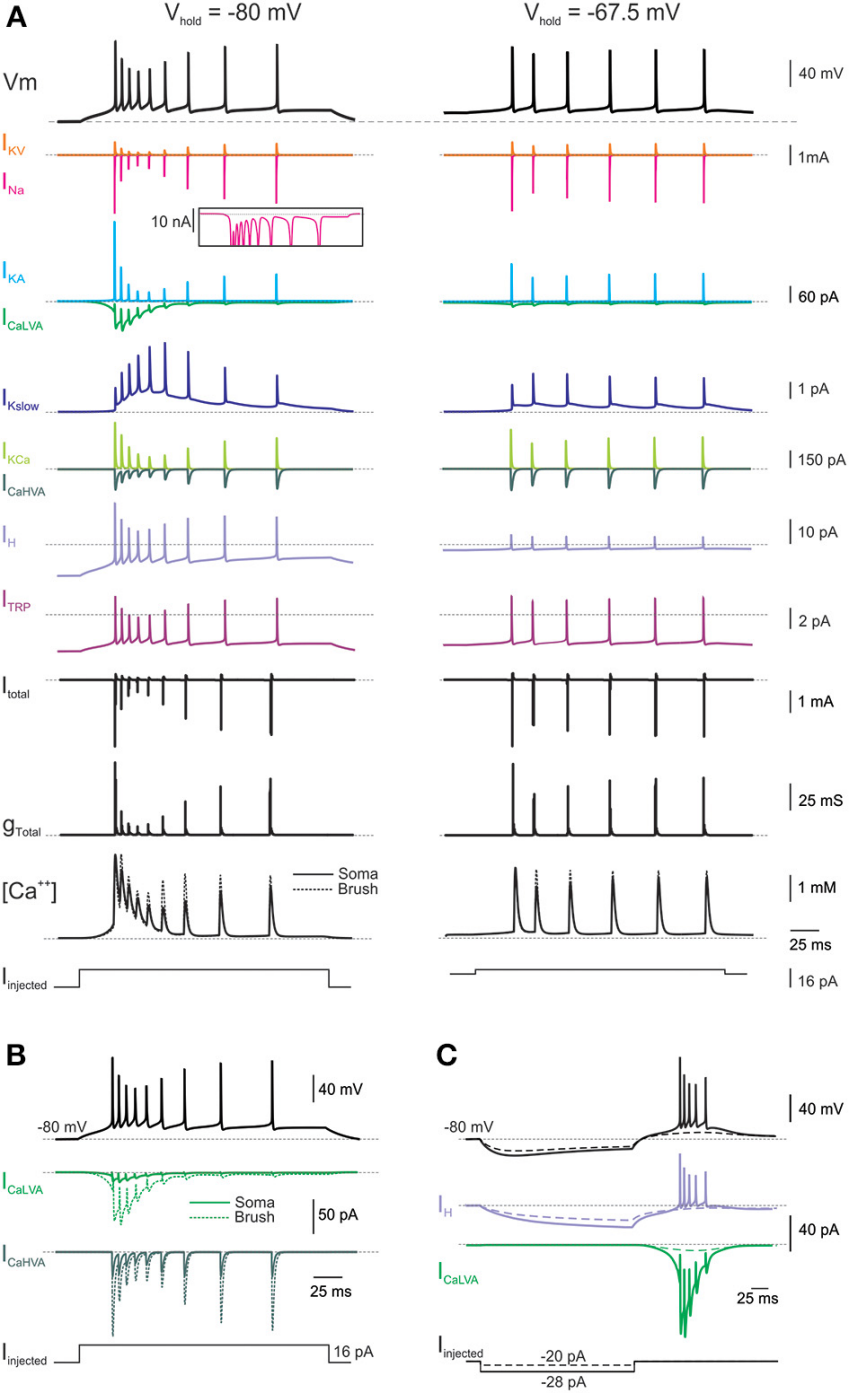
**Membrane mechanisms during repetitive firing induced by current steps. (A)** Time course of ionic currents, calcium concentration, and membrane conductance of the model caused by a burst of action potential elicited by a depolarizing current step from two different holding potentials: *left*, +16 pA from −80 mV; right, +3 pA from −67.5 mV. The inset shows a detail of the persistent sodium current component. **(B)** Calcium currents measured separately in the soma and brush compartments of the model. **(C)** Time course of ionic currents taking part to rebound burst generation in the model following −28 pA current injection from −80 mV. Names and symbols as in Methods.

**Figure 4:**
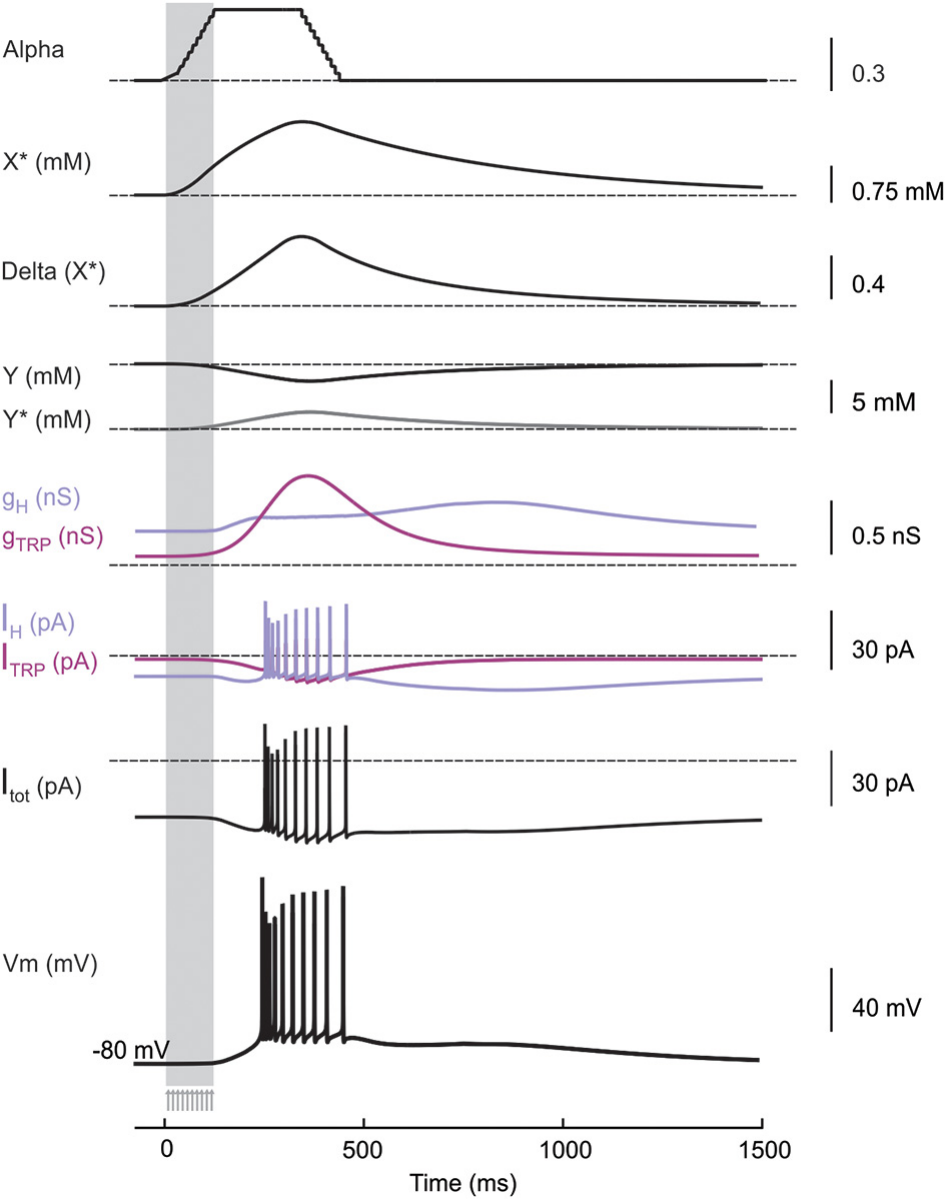
**Membrane and cytoplasmic mechanisms during LOR generation**. Time course of membrane and cytoplasmic mechanisms activated by a synaptic stimulus train (10 pulses at 100 Hz, arrows and vertical strip) delivered to the UBC model at the holding potential of −80 mV. *Alpha* is the forward rate function graded with synaptic inputs, which activates the intracellular cascade by converting the receptor *X* into X^*^. The amount X^*^ determines the conversion of the intracellular factor *Y* into Y^*^ at rate *Delta*. Y^*^ causes changes in H and TRP conductances and currents. The total current flux (*I_H_* + *I_TRP_*) triggered by the cascade mechanism depolarizes the model generating the LOR.

**Figure 5:**
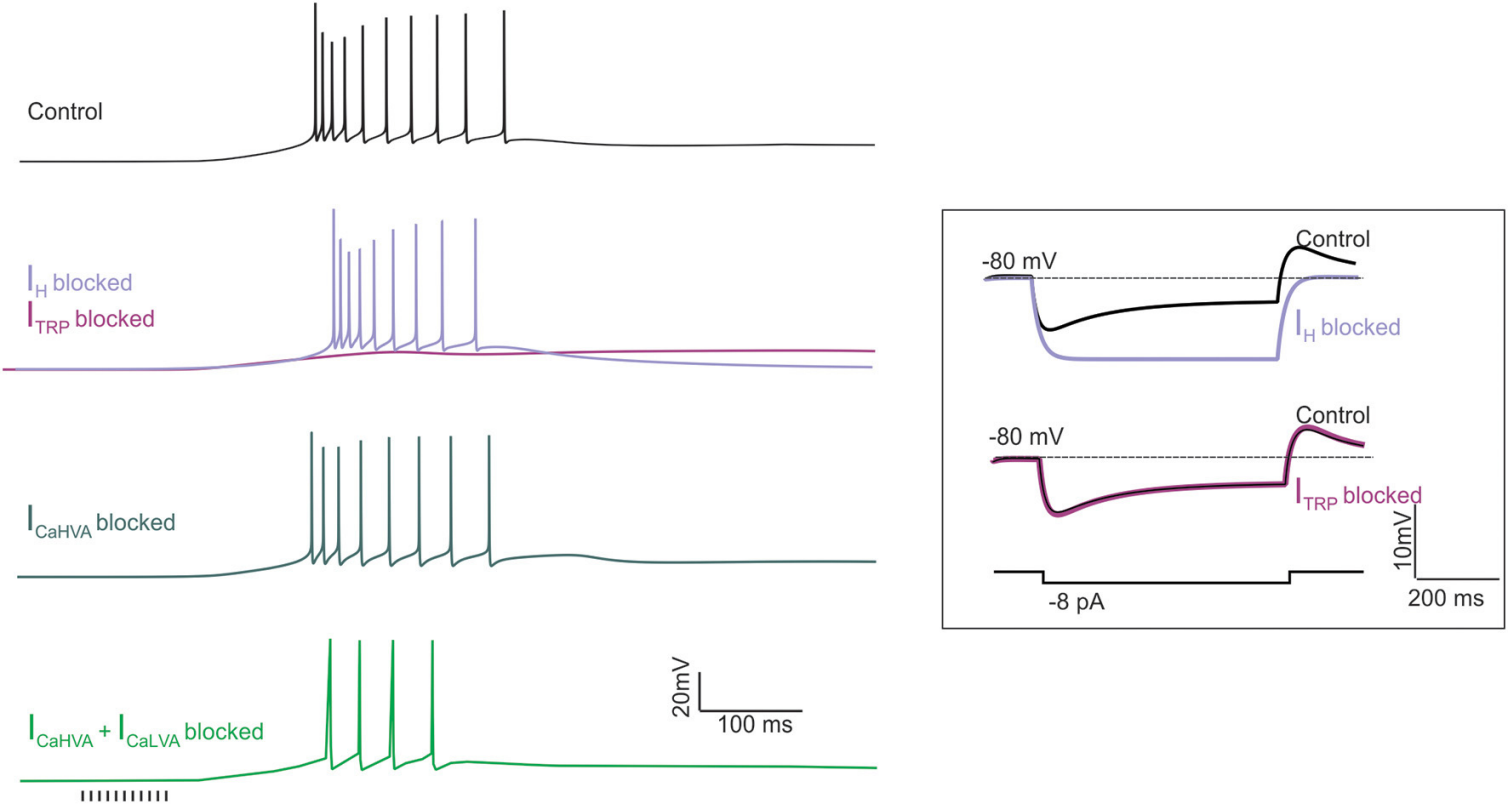
**The effect of ionic current blockage on LOR generation**. Simulations were made to mimic the pharmacological effect of ionic channel blockers on LOR generation. The LOR was elicited by a synaptic stimulus train (10 pulses at 100 Hz, arrows) delivered to the UBC model from the holding potential of −80 mV. The traces show the effect of *I_H_*, *I_TRP_*, *I_CaHVA_* and combined *I_CaHVA_* + *I_CaLVA_* block. For comparison, the effect of *I_H_* and *I_TRP_* block on membrane potential transients generated by hyperpolarizing current step injection is shown in the inset.

**Figure 6:**
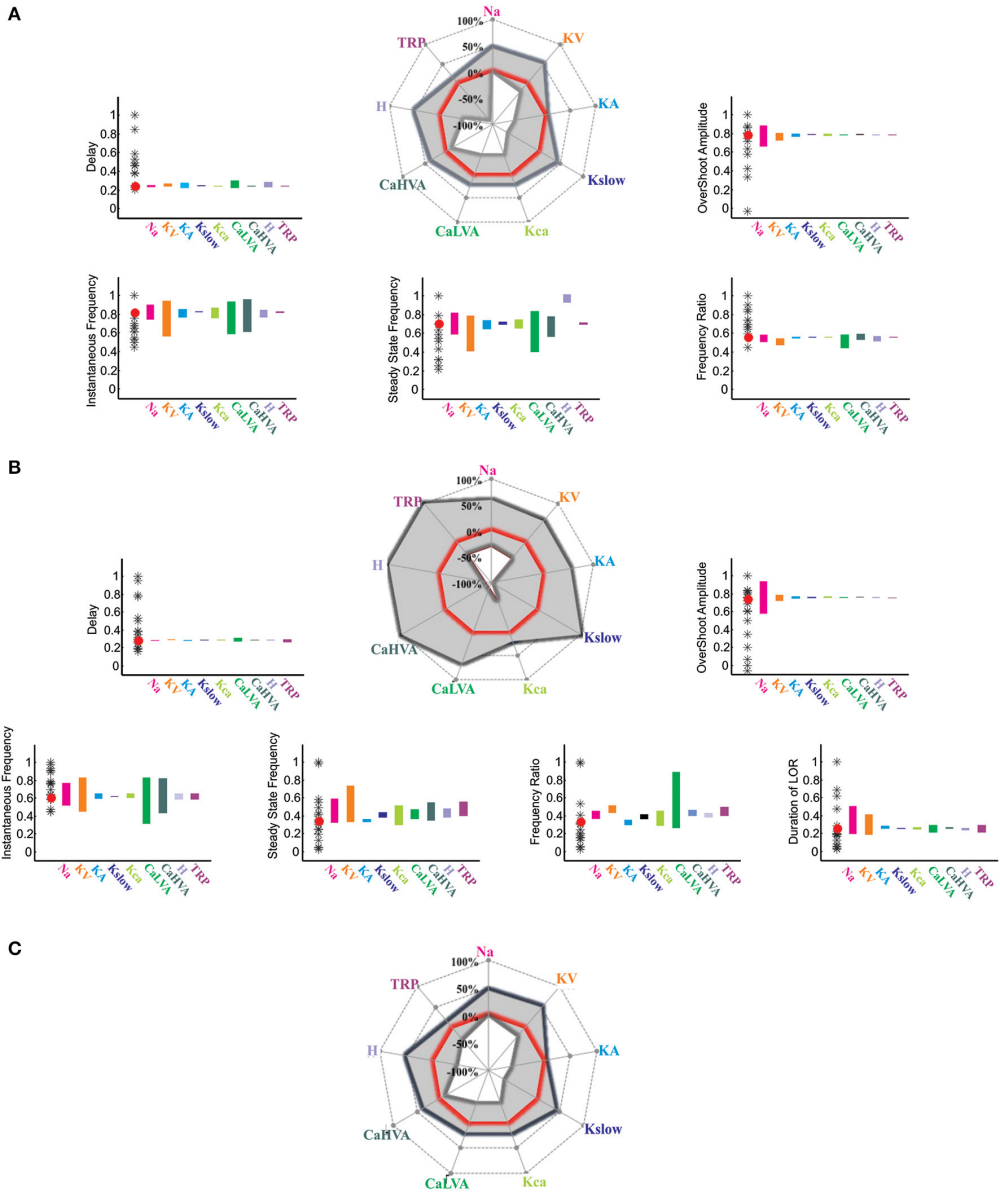
**Model robustness**. The plots illustrate the results of varying the model maximum ionic conductances between the minimum and maximum values allowing to obtain existing granule cell responses. The target parameters were spike delay, instantaneous frequency, steady-state frequency, frequency ratio (Instantaneous/steady-state frequency), spike overshoot, and LOR duration. The polar plot reports the variations observed in each of these parameter by changing the maximum conductance of each ionic channel in turn. The histograms compare model to experimental results: stars represent the experimental data, the red dot represents the response of the canonical UBC model, color bars represents the variation of each parameter while changing the maximum ionic conductance between the extremes of the variation range. **(A)** Model robustness in response to step current injection (+16 pA for 800 ms from −80 mV). **(B)** Model robustness of the LOR. **(C)** Combined model robustness for step and LOR responses.

**Figure 7:**
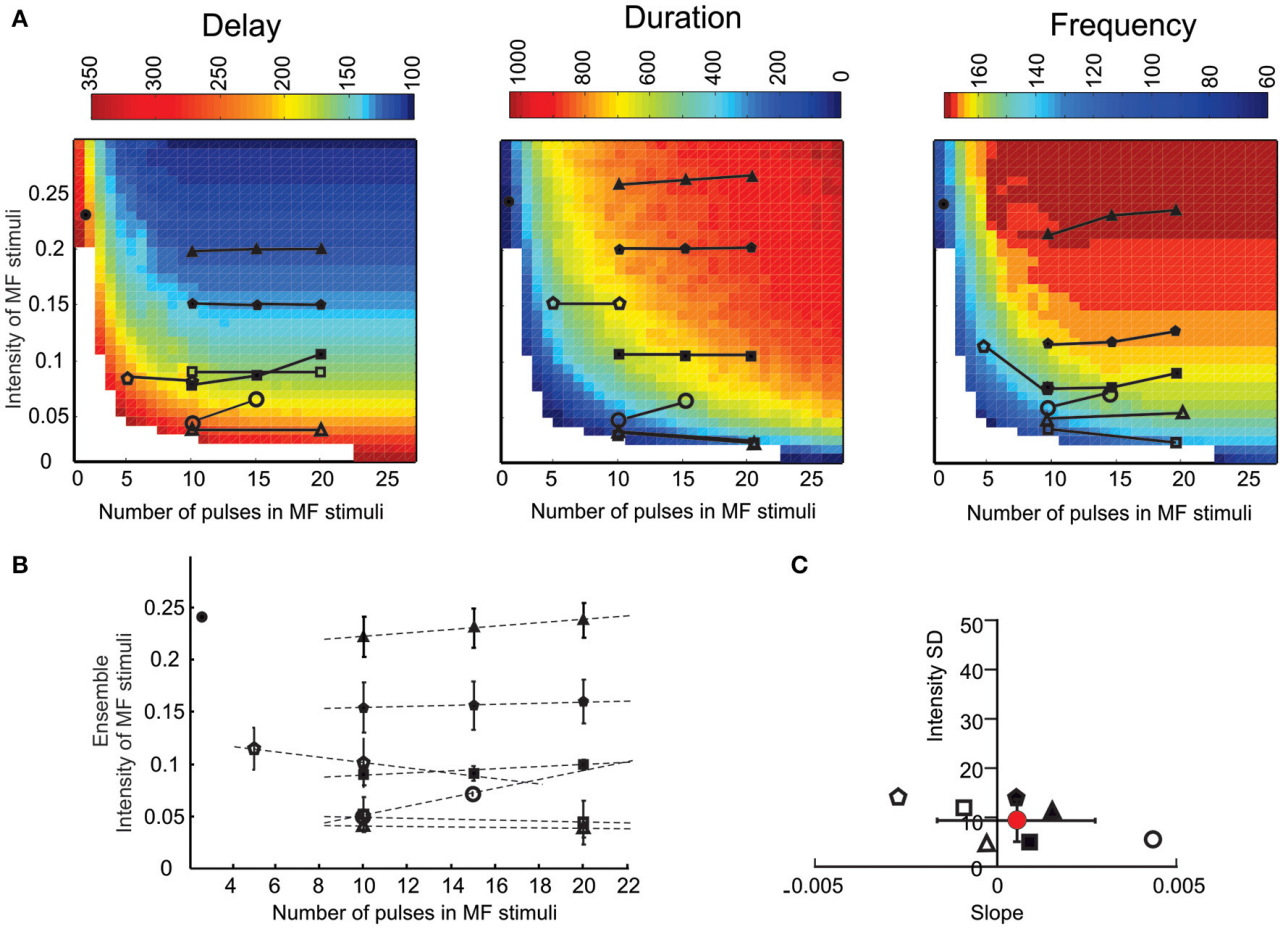
**The LOR response space**. The LOR response space was reconstructed by systematically varying the number of pulses of mossy fiber stimuli. Experimental data were reported on the LOR response space for comparison. **(A)** The three panels show the response space for spike delay, burst duration, and instantaneous frequency of the LOR. The symbols represent experimental data sets in different UBC recordings, in which the number of stimulus pulses was varied. The intensity in these plots is the dependent variable obtained at the intersection between the number of mossy fiber stimuli and the parameter values measured experimentally and reported on the response space for that specific parameter. **(B)** Ensemble intensity of mossy fiber stimuli obtained for each cell as the average of all values derived for the three plots in A (Mean ± MSE, *n* = 6 to 9 depending on the number of data points/plot). Dotted lines are linear fittings to the points of each data set. **(C)** Normalized SD of the intensity values calculated for each data set reported in **(B)** vs. the slope of the same data set.

**Figure 8:**
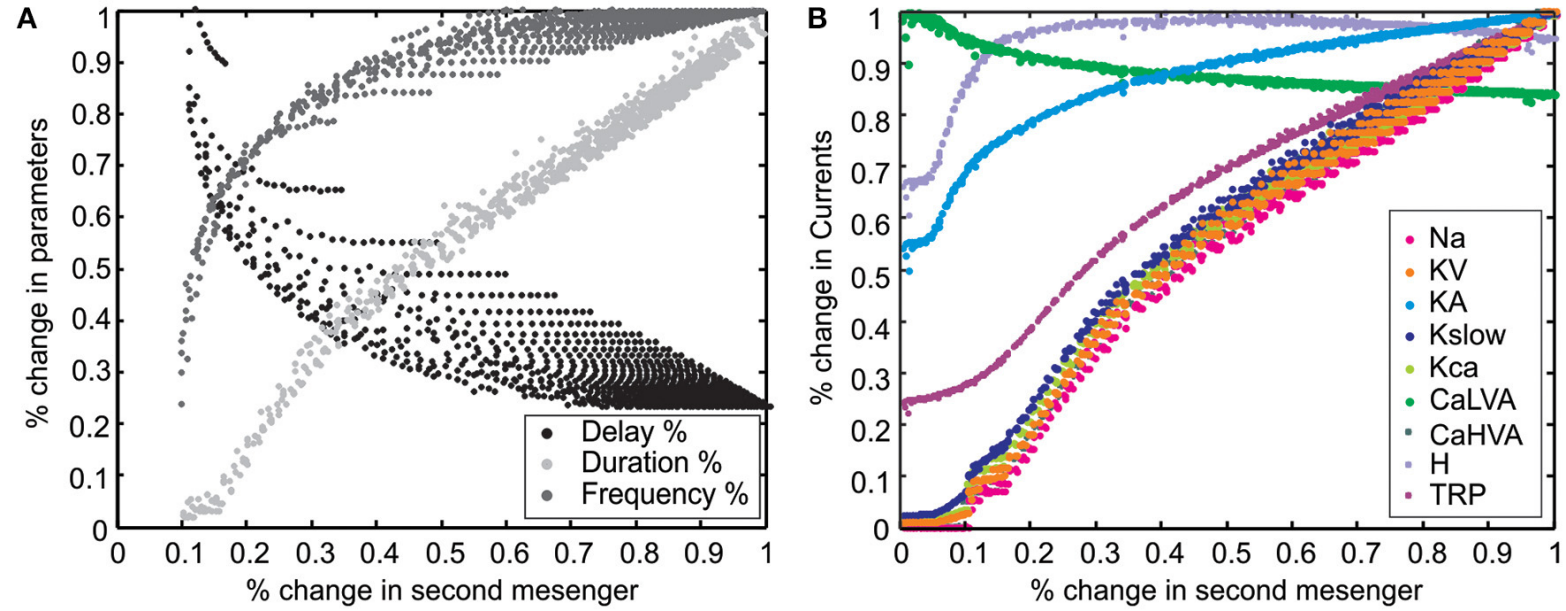
**Correlations of LOR parameters**. Variation in LOR parameters vs. second messenger concentration. **(A)** The relative change of first-spike delay, burst duration and instantaneous frequency of the LOR were plotted against second messenger concentration. The first-spike delay decreases, while the burst duration and instantaneous frequency of the LOR increase with the cAMP level. **(B)** The relative change of different ionic currents with respect to second messenger concentration. Note the specific behaviors of H, TRP, CaLVA, and KA currents.

The model was constructed respecting the morphological constraints and passive properties (Figure 1) and using the available electrophysiological data on ionic channels of UBCs (Table 1). Then the model was calibrated through a multi-parametric comparison with current-clamp response to current injection (Figures 2, 3). Finally, the model was extended through a mechanism coupling synaptic activity to ionic channel gating and became able to generate the LOR (Figure 4). The robustness of this procedure was evaluated by performing various tests allowing model predictions to be compared to native UBC responses (Figures 5–8).

The UBC model (Figure 1B) was composed of 4 main compartments (brush, dendritic shaft, soma, axonal initial segment, axon). The model was endowed with 8 voltage-gated or Ca^2+^-gated ionic currents (*I_Na_*, *I_KV_*, *I_KA_*, *I_Kslow_*, *I_KCa_*, *I_CaLVA_*, *I_CaHVA_*, *I_H_*) and with a voltage-independent leakage current (*I_TRP_*). The kinetics of individual ionic currents were fitted to UBC data (when directly available) or adapted from previous cerebellar neuron models (D'Angelo et al., 2001; Anwar et al., 2012). The density of ionic channels in different model compartments was pre-set according to the known channel localization (e.g., Na channels were more expressed in the AIS, CaLVA channels were more abundant in the brush than in the soma while CaHVA channels were equally distributed in soma and brush; (Khaliq and Raman, 2006; Diana et al., 2007) and subsequent adjustments to the current density were made to properly fit the UBC response to current injection (cf. Solinas et al., 2007a,b; see below).

The calibration of UBC passive properties was done with respect to electrophysiological measurements (Figure 1C). After having reconstructed the UBC compartmental morphology, the injection of voltage step into the model yielded input capacitance and resistance values falling in the middle of the experimental data distribution (the average experimental values were 16.3 pF and 0.97 GΩ, the average model values were 16.7 pF and 1 GΩ).

### UBC electroresponsiveness and the late-onset response (LOR)

In current-clamp whole-cell recordings, UBCs showed a typical electroresponsive pattern comprising the following elements (Figure 1A):

1. UBCs were silent at rest, with a resting potential of −66.7 ± 1.8 mV (*n* = 81) (cf. −67.6 ± 0.9 mV in Diana et al., 2007; −67.5 ± 1.4 mV in Locatelli et al., 2013).
2. During hyperpolarizing current pulses (from −80 mV), UBCs generated a sagging inward rectification followed by a rebound depolarization after the end of the pulse (Figure 2A1, left).
3. During depolarizing current pulses (from −80 mV), UBCs generated a spike burst, which, by raising current intensities, was followed by a tonic discharge (Figure 2A1, left). The spike burst was composed of a LTS surmounted by a burst of high-frequency fast spikes. An AHP appeared at the end of the spike discharge.
4. When the resting membrane potential was artificially depolarized (between −65 to −70 mV), the LTS almost disappeared and the spike burst became weaker (Figure 2A1, Right).

These electroresponsive properties were common to those reported in the previous experimental investigations (Diana et al., 2007; Birnstiel et al., 2009; Locatelli et al., 2013), in which the implication of specific ionic channels was examined (see below).

Following mossy fiber bundle stimulation in the presence of GABA and glutamate receptor antagonists, UBCs showed a LOR composed of a depolarizing ramp followed by a LTS surmounted by a high-frequency spike burst followed by a prolonged tonic discharge (Figure 2B1). The LOR corresponded to the same phenomenon reported by Locatelli et al. (2013), who suggested that the LOR depended on a metabotropic mechanism involving cAMP increase in the cytoplasm, leading to the H- and TRP-channel modulation. Notably, the delay, duration and frequency of the LOR were apparently related to the intensity and number of pulses used for mossy fiber bundle stimulation (Figure 2B1). Higher intensity or number of pulses caused shorter delay, higher frequency, longer duration of the LOR (quantitative data are given below in Figures 6, 7).

The UBC model was parameterized against responses to step-current injection (Figure 2B1). The parameters related to spikes elicited by *depolarizing current steps* (e.g., using 16 pA from a holding potentials of −80 mV) included spike delay (30.3 ms), spike overshoot (36.7 mV), instantaneous frequency (176 Hz), steady state frequency (126 Hz), and frequency ratio (0.715). The parameters related to *hyperpolarizing current steps* included sag amplitude (e.g., −17.3 mV with a −20 pA step from −80 mV) and current threshold for rebound bursting (e.g., −20 pA from −80 mV). The match of model to the experimental data was statistically significant (Student's *t*-test, *p* < 0.01) for all the parameters. This model, once endowed with coupling mechanisms regulating TRP and H channels, predicted the LOR following synaptic stimulation (Figure 2B2).

It should be noted that what has been developed in this paper is a *canonical* model matching the properties of an ideal UBC. Variants in terms of burst length, frequency ratio, or rebound depolarization observed in some UBCs could all be obtained through slight changes of maximum current densities for specific ionic channels (data not shown).

### UBC electrogenic mechanims in the model

In the model, as in real UBCs, a small depolarizing current (8–16 pA) from a negative potential (−80 mV) was sufficient to trigger the LTS and a short spike burst, while a larger depolarizing current (>16 pA) was required to drive the model into tonic discharge (Figures 2B1, 3A; cf. Locatelli et al., 2013). However, when depolarizing step-current injection was applied from resting potential (−67.5 mV; Diana et al., 2007; Locatelli et al., 2013; Figure 2B1, Left), the response was characterized by a tonic discharge almost lacking the initial spike bust (Figure 2B1, Right). Even by using a net current injection stronger than that used from −80 mV, the instantaneous spike frequency was apparently lower. While a classical set of currents—including *I_Na_*, *I_KV_*, *I_KA_*, *I_CaHVA_*, and *I_KCa_*—was sufficient to determine the spike shape as well as spike frequency and its variations (D'Angelo et al., 2001), specific ionic channels were required to determine these characteristic properties of UBC electroresponsiveness.

The high responsiveness of the UBC model to small current injection from negative holding potentials was due not only to the relatively high input resistance but also to activation of the CaLVA current. CaLVA was the most prominent current in this functional regimen and regeneratively amplified the effect of current injection causing the LTS. CaLVA currents have a voltage-dependent inactivation, reflected into the marked weakening of bursting elicited from more depolarizes membrane potentials (Diana et al., 2007; Birnstiel et al., 2009; Locatelli et al., 2013). The density of CaLVA currents was derived from voltage-clamp recordings (Diana et al., 2007) and, together with a precise setting of input resistance, provided a major constraint for calibrating the whole electroresponsive mechanism. Once the LTS was generated, the depolarization gated the other ionic channels so that various currents contributed to generate the spike bursts and control its evolution. As in real UBCs, burst spike frequency could raise up to 200 Hz along with spike amplitude adaptation. The burst was probably enhanced by the resurgent Na current (Raman and Bean, 1997; Afshari et al., 2004; Dover et al., 2010) and the first spike delay was regulated by *I_KA_*.

With depolarizing current injections <16 pA, the burst was driven by the LTS and terminated after a time compatible with CaLVA channel inactivation. With depolarizing current injections >16 pA, spike discharge protracted beyond the burst through the intervention of another set of ionic channels: the CaHVA current and the persistent Na current sustained a depolarizing plateau maintaining spike discharge. The model predicted that an appropriate balance between the CaHVA and a slow K current, Kslow, was required to regulate the intensity and duration of this protracted spike discharge. Finally, during depolarization, the H current was deactivated and therefore reduced the total input conductance favoring depolarization, but its contribution to the total UBC current was limited by the decreased driving force.

As in real UBCs, when injected with hyperpolarizing current steps, the model generated sagging inward rectification followed by rebound excitation after the end of the pulse. The rebound could be consisted of a depolarizing ramp that could prime a LTS and a spike burst when the hyperpolarizing was more intense and protracted (Figure 3C). The rebound was driven by H current deactivation and amplified by CaLVA current activation (see Figure 6; Russo et al., 2007; Locatelli et al., 2013). The H current was derived from voltage-clamp recordings (Locatelli et al., 2013) and provided a critical constraint for model calibration.

### Modeling the late-onset response

By using the model, we tested the hypothesis that synaptic modulation of H- and TRP-currents through an intracellular cascade was *necessary and sufficient* to generate the LOR (Locatelli et al., 2013). Therefore, if the UBC model was correctly constructed, it should be able to generate the LOR by coupling synaptic transmission to H- and TRP-current gating through an appropriate second messenger pathway. The second messenger pathway was implemented as a generic reaction scheme, in which (1) mossy fiber bundle stimulation activated the extra-synaptic receptor *X* to X^*^, (2) X^*^ increase above 60% of its maximum value initiated conversion of a second messenger *Y* to Y^*^, and (3) Y^*^ shifted the voltage-dependence of *I_H_* and the maximum conductance of *I_TRP_* (see Methods; DiFrancesco and Mangoni, 1994; Locatelli et al., 2013; Figure 4). The increased Y^*^ concentration eventually caused a proportionate increase in TRP maximum conductance and a proportionate shift of *I_H_* activation toward positive membrane potentials. TRP depolarized the membrane sufficiently to generate the LOR, while *I_H_* transiently unbalanced the sub-threshold current generating a slow depolarizing ramp tuning the delay of LOR initiation (Figure 4). It is worth noting that, after setting model intrinsic electroresponsiveness, no further changes was required to obtain the LOR, which is therefore a true model prediction.

### Quantitative comparison of the model with experimental data

The model could reproduce the fundamental properties of the LOR, which are observed in WCR recordings (this work and Locatelli et al., 2013). (i) The LOR was dependent on the intensity and number of synaptic pulses (cf. Figure 2). (ii) The TRP current generated the major depolarizing drive for LOR generation, while the H-current regulated LOR delay by controlling the slope of the depolarizing ramp. Blocking H- and TRP-currents together abolished the LOR (Figures 4, 5). (iii) The Ca^2+^ currents enhanced LOR spike frequency, with a more evident contribution given by LVA than HVA currents. However, Ca^2+^ currents did not regulate the depolarizing ramp nor they were required to generate the LOR itself (Figure 5). (iv) The LOR was poorly affected by holding potential and could be generated either from −80 or −67.5 mV (data not shown). The reliability of model predictions about UBC electroresponsiveness and LOR generation were assessed through a quantitative comparison with experimental data.

*I_H_* provided a critical constraint for the model and the effect of pharmacological block by ZD7288 and Cs^+^ has been reported (Diana et al., 2007; Russo et al., 2007; Locatelli et al., 2013). *I_H_* switch-off in the model caused hyperpolarization of resting membrane potential (Figure 5) and eliminated the sag during hyperpolarizing current steps (Figure 5, inset). The resting membrane potential shift (−4 mV) and differences between the maximum voltage deflection and the voltage attained at the end of the current step (from 3.96 to 0.13 mV) and the subsequent rebound depolarization (from 2.54 to 0.15 mV) were consistent with previous experimental data (Locatelli et al., 2013). Finally, the LOR delay increased by 12.8% and the slope of the depolarizing ramp and its duration decreased by 12.2 and 24.9%, respectively, again reflecting experimental data (Locatelli et al., 2013). Therefore, in the model the *I_H_* regulated the delay of the late-onset burst by controlling the depolarizing ramp similar as in whole-cell recordings.

The TRP channels provided a background leakage current, which increased during the LOR. *I_TRP_* switch-off in the model prevented full LOR generation leaving only a subthreshold depolarization driven by the H current (Figure 5). The slope of the depolarizing ramp decreased by 69%, consistent with 49% decrease measured experimentally following channel blockage with SKF96365 (Locatelli et al., 2013).

Pharmacological blockage of CaLVA channels (with mibefradil) and CaHVA channels (with nimodipine) was reported to modify spike firing in the LOR but not to prevent LOR generation (Locatelli et al., 2013). In the model, CaLVA switch-off did not prevent the LOR but reduced the number of spikes by 20% and the instantaneous frequency by 38%. Combined CaHVA and CaLVA currents switch-off did not prevent the LOR but reduced the number of spikes by 80% and the firing frequency by 88% (Figure 5). These effects in the model are comparable to those of pharmacological blockage reported experimentally.

### Robustness of model predictions

As explained above (see also Methods), reference values were obtained from UBC voltage-clamp data on the H- LVA- HVA- and TRP-currents and were then used to pre-set the corresponding maximum conductance values in the model. The other maximum conductance values were set respecting proportions evaluated in previous models [for example the Gmax (*I_Na_*)/G_max_ (*I_KV_*) ratio, the G_max_ (*I_KCa_*)/G_max_(*I_CaHVA_*] ratio, (Gabbiani et al., 1994; D'Angelo et al., 2001; Solinas et al., 2007b). Nonetheless, the maximum ionic conductances in the model were actually free parameters, as they were tuned to match UBC electroresponsiveness. A robustness analysis was then performed in order to estimate the confidence intervals of the maximum ionic conductances still allowing to generate existing UBCs. The range of variations allowed for each maximum conductance were used to maintain the model within the experimental values of first spike delay, instantaneous frequency, steady-state frequency, frequency ratio and spike overshoot (Figure 6). Finally the model predicted, through the cytoplasmic coupling mechanism, the experimental values measured in the LOR for the parameters indicated above as well as for LOR duration.

The experimental parameters measured in the UBCs covered in this study were used to set the range limits for parameter variation in the model. *Intrinsic excitability* was characterized by spike delay (31.1–93.4 ms, *n* = 12), spike overshoot (15.0–45.1 mV, *n* = 12), instantaneous frequency (95.9–202.7 Hz, *n* = 12), steady-state frequency (41.2–141.8 Hz, *n* = 12), and frequency ratio (0.61–1.3, *n* = 12). The *LOR* was characterized by spike delay (98–1000 ms, *n* = 18), spike overshoot (10.2–44.5 mV, *n* = 18), instantaneous frequency (92–209 Hz, *n* = 18), steady-state frequency (19.5–60 Hz, *n* = 18), frequency ratio (0.1–0.6, *n* = 18), and burst duration (100–2500 ms, *n* = 18). Simulation results were accepted as possible UBC responses when falling within these limits.

Both concerning responses to current injection (Figure 6A) and the LOR (Figure 6B), the modulation of maximum ionic conductances led within boundaries spanning ≤ ± 20%. By combining the two sets of boundaries obtained for current injection and LOR gave the compound boundaries (Figure 6C). This robustness analysis showed that the canonical model was well balanced and could use the same ionic channel settings to faithfully explain both electroresponsiveness and LOR.

### Prediction of late-onset response activation by different activity patterns

The model allowed to predict burst delay, duration and frequency of LOR in relation to different combinations of stimulus duration and intensity (Figure 7A). The response showed opposite changes for delay (which decreased with stimulus duration and intensity) compared to duration and frequency (which increased with stimulus duration and intensity). If the simulated response space was correct, the experimental data points obtained changing stimulus duration should actually correspond to the same stimulus intensity. Actually, for all parameters, the experimental data points fell on nearly horizontal lines (Figure 7A). A quantitative assessment of the matching between experiments and model was obtained by calculating the slope of the duration/intensity relationships (Figure 7B) and by assessing the variability of the mean predicted stimulus intensity (Figure 7C). The slope of the duration/intensity relationships was 0.0005 ± 0.002 and was statistically indistinguishable from 0 (*p* < 0.001, *n* = 7, *t*-test). The variability of the predicted stimulus intensity was <10%. Therefore, the model could effectively predict LOR properties while varying the stimulus pattern.

### Prediction of mechanisms determining LOR properties

The explanation of why, in the LOR stimulus/response space, similar parameter values were determined by varying either stimulus duration or intensity (cf. Figure 8A) had to be searched in their control over the common factor, Y^*^. In turn, Y^*^ controlled membrane channel gating and therefore the LOR (Figure 8B).

The effect of Y^*^ on LOR parameters (burst delay, duration and frequency) for all possible combinations of stimulus duration and intensity used in Figure 7 are shown in Figure 8A. In particular, while increasing Y^*^, burst delay decreased almost exponentially, burst frequency increased almost exponentially, and duration increased almost linearly (Figure 8A; the presence of multiple points at each Y^*^ value reflected the different Y^*^ kinetics for different combinations of stimulus intensity and duration).

A mechanistic explanation of the LOR properties was obtained by correlating Y^*^ with the underlying ionic currents (Figure 8B). It turned out that the Y^*^ dependence of *I_Na_*, *I_KV_*, *I_Kslow_*, *I_CaHVA_*, *I_KCa_*, and *I_TRP_* was similar to that of LOR duration. The Y^*^ dependence of *I_H_* and *I_KA_* was similar to that of LOR frequency and opposite to that of LOR delay. The Y^*^ dependence of *I_CaLVA_* was inverted compared to any others. This result implies a complex interaction of all currents in regulating LOR properties and a primary role of *I_H_* in controlling LOR delay.

## Discussion

This paper shows that regulation of H- and TRP-currents by an intracellular factor can generate the LOR, a slow depolarization driven by synaptic activity recently observed in cerebellar UBCs. The LOR mechanism was explained by a model accounting for UBC electroresponsiveness and including cytoplasmic coupling to H- and TRP channels. Compared to glutamatergic synaptic responses, the LOR generated a spike burst with longer delay, which was modified by the pattern of mossy fiber discharge. The LOR, together with specialized mechanisms of intrinsic electroresponsiveness and synaptic transmission, endows UBCs with the ability to generate spike bursts with variable delay in the cerebellar granular layer.

### The UBC canonical model: properties and limits

The present model provides a *biologically realistic reconstruction* of UBC electroresponsiveness (Diana et al., 2007; Russo et al., 2007; Birnstiel et al., 2009; Locatelli et al., 2013) based on the representation of neuronal geometry, passive properties and on the subsequent insertion of appropriate ionic and intracellular mechanisms. The H, TRP, and Ca^2+^ conductances provided the critical constraints for UBC modeling and had density, gating and kinetics derived from UBC recordings (Diana et al., 2007; Russo et al., 2007; Birnstiel et al., 2009; Locatelli et al., 2013). These core mechanisms were associated with a spike generation mechanism sustained by Na^+^ and K^+^ conductances. The CaLVA and CaHVA channels were distributed across soma, dendrite and brush in order to generate calcium currents proportional to those determined experimentally (Diana et al., 2007; Birnstiel et al., 2009), while the Na channels were localized in soma, initial segment and axon. The localization of ionic channels had an impact on model electrogenesis. On one side, the CaLVA current was larger in the brush than soma, while the CaHVA current was equally distributed, as indicated by calcium current measurements (Diana et al., 2007; cf. Figure 3C). The high density of CaLVA channel in the brush, coupled with limited current flow to the soma through the dendrite, enhanced the local auto-regenerative LTS depolarization. This would become particularly effective when synaptic currents are injected into the brush. On the other side, the Na^+^ current was concentrated in the soma, initial segment and axon, conferring higher excitability and normalizing spike and burst discharge parameters (not shown). The precise localization of Na^+^ channels in UBCs remains to be determined by immunohistochemistry.

The resulting model was a *canonical* (or prototypical) representation of the UBC, such that ±20% variations of ionic channel maximum conductances still allowed model responses (first spike delay, initial firing rate, steady-stated firing rate, frequency ratio, spike overshoot) to remain within the range of parameters recorded experimentally. This variation range is normally observed while measuring ionic currents in neurons (Bhalla and Bower, 1993) and has also been reported in UBCs for H-, CaLVA, and CaHVA currents (Diana et al., 2007; Birnstiel et al., 2009; Locatelli et al., 2013). Most importantly, the model allowed to predict the LOR without any further modifications, provided that the cytoplasmic coupling mechanism was properly implemented.

Beside its effectiveness, some aspects of the UBC model were admittedly simplified. (i) The H-current kinetics observed in UBC recordings (Locatelli et al., 2013) suggest that H-current gating could reflect differential expression and assembly of subunits. This could contribute to tune UBCs toward specific LOR kinetics. (ii) The Na current was implemented using a 13-state stochastic model for Nav1.6 channels of granule cells generating transient, persistent and resurgent current components (Raman and Bean, 2001; Magistretti et al., 2006; Dover et al., 2010). While these three components are present in all cerebellar neurons (Afshari et al., 2004; Khaliq and Raman, 2006), the resurgent current may be proportionally higher in UBCs than granule cells (Afshari et al., 2004). This could further promote the initial spike burst primed by the LTS. (iii) The regulation of duration and frequency of tonic discharge following the LTS required that a slow K current, Kslow(D'Angelo et al., 2001), was added to the model. Moreover, the regulation of first-spike delay required that a fast-activating K current, KA(D'Angelo et al., 2001), was added to the model. These current have not been reported in UBCs yet and are therefore model predictions, which remain to be proved experimentally. (iv) Intracellular calcium regulation was modeled using a simplified scheme useful to couple calcium entering through CaHVA channels to BK-type channel gating and the effect of more complex schemes remains to be explored.

In a further evolution, the model may benefit of a detailed morphological reconstruction of UBC brush and axon along with proteomic and channelomic analysis directly defining the molecular properties of ionic channel subtypes and of the cytoplasmic cascade coupling membrane receptors to ionic channels. It will also be of interest to determine under which circumstances routines based on genetic algorithms will be able to solve the complex optimization problem of the UBC model (Druckmann et al., 2007, 2008, 2011, 2013).

### The three core mechanisms of burst generation in UBCs

A quite relevant property of the UBC model, as well as of real UBCs, was that of generating a variety of burst responses, which were controlled by the intervention of H-, TRP-, and Ca^2+^- currents. The model showed that the interaction of H- TRP-, and Ca^2+^- currents emerged in three conditions.

1. *Bursts during depolarization from negative membrane potential*. In response to depolarizing current injection, the *I_CaLVA_* was critical to promote spike generation.
2. *Bursts at the end of a hyperpolarization*. Following a hyperpolarization, *I_H_* caused a rebound excitation boosting the voltage-dependent activation of *I_CaLVA_* and promoting generation of rebound bursting.
3. *Bursts during the LOR*. During the LOR, the ramp was promoted by *I_TRP_* and regulated by *I_H_*. *I_CaLVA_* then amplified the depolarization intensifying the subsequent spike burst.

These mechanisms, especially the first two, proved markedly voltage-dependent due to the specific voltage-dependent inactivation of CaLVA channels. It is therefore predicted that alternating cycles of depolarization and hyperpolarization involving the granular layer inhibitory circuit (Vos et al., 1999) could modify the ability of the UBCs to generate spike bursts substantially.

### LOR generation in the model

Rather than reproducing the intracellular biochemical pathway (which remains largely undetermined) we have developed a generic coupling mechanism including receptors activated by synaptic activity and leading to the production of a second messenger (Y^*^), possibly corresponding to ATP/cAMP conversion. The level of this second messenger caused the current influx (~-16 pA) generating the depolarizing ramp and the LOR, with a delay depending on the second messenger concentration and ranging up to several hundreds of milliseconds.

Thus, the model provided substantial support to the existence of an intracellular mechanism coupling membrane receptor activation to H- and TRP-channels and causing the LOR in UBCs. Modeling suggests that *I_H_* was responsible for controlling LOR delay and frequency, that *I_CaLVA_* and *I_KA_* were driven into the control of LOR delay and frequency through their time- and voltage-dependent inactivation, that *I_Na_*, *I_CaHVA_* and *I_TRP_* currents drove the depolarization during the burst and determined burst duration and activation of *I_KV_*, *I_Kslow_*, and *I_KCa_*, which actually regulated the frequency and terminated the burst.

### The LOR and delay-lines in the cerebellum

Through the LOR, UBCs can translate the intensity of the input (coded as the number of active fibers and duration of their discharge) into output bursts with different delay, duration and frequency. This implements a time-code that could reverberate through the network along the chains made by UBCs with other UBCs and granule cells (Nunzi and Mugnaini, 2000; Nunzi et al., 2001). The LOR could be modulated by local network activity causing various patterns of UBC excitation and inhibition (Ruigrok et al., 2011; Rousseau et al., 2012). Recently, a frequency-dependent regulation of AMPA receptor-mediated after responses has also been reported (van Dorp and De Zeeuw, 2014) and has been suggested to provide a system to generate protracted responses in UBCs. The implementation of these AMPA receptor-dependent mechanisms into the UBC model would allow to test its actual impact on UBC responses and its relationship with the LOR.

It has been proposed that UBCs are essential for shifting and converting the phase of mossy fiber activity that relays information from the vestibular apparatus, eyes, or neck, and that their characteristic cellular properties are particularly relevant for controlling and consolidating motor learning in paradigms such as VOR phase reversal (Gao et al., 2012). Actually, the LOR could help implementing the “velocity storage” system (Dai et al., 2007), which allows the vestibulo-cerebellum to transform the head velocity signal into commands for ocular motor neurons controlling the slow phase of nystagmus (Goldberg et al., 1984; Okada et al., 1999). Patterns of activity compatible with the intervention of the LOR in UBCs have been reported in the electric organ (a cerebellar-like structure) of mormyrid fishes during the prediction of the sensory consequences of motor acts (Kennedy et al., 2014). By involving intracellular cascades, the LOR could be modified by receptor systems—involving e.g., acetylcholine, serotonin, and noradrenaline—known to modulate H and TRP channels, thus correlating cerebellar computation with the brain functional state.

## Conclusions

The UBCs, despite their relatively recent discovery, are revealing specific cellular mechanisms generating a rich set of electroresponsive properties. By these means, UBCs generate different types of burst responses including the LOR. The LOR endows the granular layer with a long-sought slow process extending the timing capabilities of the cerebellar network (Dean and Porrill, 2008). The model predicts that the H-current and the TRP-current are critical to generate the LOR through membrane receptors and cytoplasmic cascades that remain to be elucidated. The model also predicts the specific roles for the different types of Ca^2+^ and Na currents observed in UBCs and predicts the existence of fast and slow-K currents (of the category of *I_KA_* and *I_Kslow_*), which remain to be demonstrated experimentally. The UBC model provides a useful new tool for investigating granular layer spatio-temporal dynamics and for implementing large-scale network models of the cerebellum.

### Conflict of interest statement

The authors declare that the research was conducted in the absence of any commercial or financial relationships that could be construed as a potential conflict of interest.
